# Species Diversity Effects on Productivity, Persistence and Quality of Multispecies Swards in a Four-Year Experiment

**DOI:** 10.1371/journal.pone.0169208

**Published:** 2017-01-03

**Authors:** Jingying Jing, Karen Søegaard, Wen-Feng Cong, Jørgen Eriksen

**Affiliations:** Department of Agroecology, Aarhus University, Tjele, Denmark; Institute of Tibetan Plateau Research Chinese Academy of Sciences, CHINA

## Abstract

Plant species diversity may benefit natural grassland productivity, but its effect in managed grassland systems is not well understood. A four-year multispecies grassland experiment was conducted to investigate the effect of species diversity–legumes and non-leguminous forbs–on productivity, persistence and sward quality under cutting or grazing regimes and with or without slurry application. Three mixtures were established– 3-mix: grass, red and white clover, 10-mix: 3-mix plus birdsfoot trefoil and six non-leguminous forbs, and 12-mix: 10-mix plus lucerne and festulolium. Species diversity increased sward production and yield persistence under cutting regime. The 12-mix had the highest yield from the second year onwards and no statistically significant yield reduction over four years, while annual yields in the 3-mix and 10-mix decreased significantly with increasing grassland age. The higher yield in the 12-mix was mainly due to the inclusion of high-yielding lucerne. The 10-mix and 12-mix had lower proportions of unsown species than the 3-mix, the difference being dependent on grassland age. Generally, the 3-mix had higher concentrations of in-vitro organic matter digestibility (IVOMD), neutral detergent fiber (NDF) and crude protein (CP), and a lower concentration of ash than the 10-mix and 12-mix. Slurry application increased annual yield production by 10% and changed the botanical composition, increasing the proportion of grass and decreasing the proportion of legumes. Compared to cutting, grazing increased forage production by 9% per cut on average and lowered legume and forb proportions in the mixtures, but yields did not differ among the three mixtures. Overall, our results suggest that species diversity increases sward productivity and persistence only under an ungrazed cutting regime. We conclude that increasing species diversity by selecting appropriate species with compatible management is key to achieving both high yields and high persistence in managed grasslands.

## Introduction

Plant diversity has often been shown to increase productivity and stability in natural grasslands [1–4]. The productivity benefit of a diversified ecosystem has been attributed to either species complementarity where resources are used more efficiently by a greater number of species, or to the promotion of certain species with superior traits due to a selection or sampling effect [5]. Thus, there is a growing interest for exploring the potential of a higher species diversity to promote forage production, persistence and nutritive value in European agricultural grasslands. Previous studies have shown contrasting effects of plant diversity on forage yield, ranging from positive effects [6–9] to no effects [10], or even negative effects [11]. A primary conclusion drawn from these contrasting results is that the effect of species diversity on forage production *per se* is not as important as that of certain plant functional groups and/or the combination of these functional groups [12], probably because functional trait diversity and composition in complex forage mixtures can be a strong driver of resource utilization and yield production [13].

Grass-legume mixtures such as ryegrass-clover swards are commonly used in temporary agricultural grassland, because the system has the ability to be nitrogen self-sufficient and to deliver economically nutritious forage legumes [14]. Recent studies have identified a number of dicotyledonous plant species with a high forage productivity when grown in complex forage communities [15, 16]. For example, chicory (*Cichorium intybus* L.) is a perennial deep-rooting, broad-leafed forage forb documented as a valuable forage species with high productivity and high feed value [17–19]. Plantain (*Plantago lanceolate* L.) is another forage forb characterized by deep roots, drought resistance and a wide distribution in temperate grasslands [20, 21]. Lucerne (*Medicago sativa* L.) is a widely grown perennial forage legume also with a large and deep rooting system which has been cultivated in temperate and subtropical areas for forage and grazing [22]. The three dicotyledonous species are thus complementary in resource utilization in deeper soil layers when grown with grass-clover mixtures, and have potential for further improving herbage yield production in temporary grasslands.

Extending the duration of temporary grassland has been demonstrated to have beneficial environmental effects such as enhancing soil organic matter content and reducing greenhouse gas emission [23]. Furthermore, there could be economic benefits from a longer-term sward due to reduced costs of reseeding. The persistence of the sward may be improved by including more persistent species and by improving management. However, there is still scant knowledge about the effect of increasing species richness by using highly persistent species on the durability of multi-species grasslands.

Previous studies have shown contrasting effects of grazing versus cutting on herbage production. For example, higher herbage yield under cutting compared to grazing were observed by Binnie and Chestnutt [24] and Jackson and Williams [25]. In contrast, Creighton et al [26] found no difference in herbage production between grazing and cutting; Lantinga et al [27] found 10% higher herbage production in animal-grazed swards compared to cutting swards at 250 kg N ha^-1^, whereas the difference disappeared at 550 kg N ha^-1^. These contrasting effects were probably attributed to differential effects of grazing and cutting on plant species, consequently influencing sward structure [28], productivity and persistence. To date, there is lack of comparative study on how cutting versus grazing regulates the effect of forage species diversity on productivity and botanical composition in species-diverse forage communities with dicotyledonous plant species.

The plant species composition of grasslands greatly influences the nutritive value of the herbage. Increased plant species diversity has been found to result in greater stability of plant community biomass but not the variability of individual plant species abundance [2]. In mixed species swards, variation in nutritive value may increase with higher species diversity because of inherent differences in chemical composition and different stages of maturity in the plant community [29, 30]. Thus, we hypothesized that by increasing species diversity the changes in botanical composition may influence the nutritive value of complex forage mixtures.

The aim of this study was to determine the effects of forage diversity–via forbs and persistent species–on productivity, persistence and the nutritive quality of swards in a four-year multispecies grassland experiment. Specifically, the objectives were to: (1) investigate the effect of species diversity on annual yield and persistence, botanical composition and nutritive value with/without slurry application under a cutting regime over a four-year period and (2) compare the effect of forage diversity on productivity and botanical composition between the cutting and grazing regime.

## Materials and Methods

### Experimental setup

A field experiment was conducted on a loamy sand at the Foulumgaard Research Farm in Denmark (9°34’59 E, 56°29’22 N). Permits for conducting field work were obtained from Aarhus University. This experiment was carried out over four years in an organic grass-arable system [31] which has been managed according to EU organic farming standards since 1987. Grasslands of different ages were established by progressively undersowing in spring barley (*Hordeum vulgare* L.) in four consecutive years, 2006–2009. Thus, ages of grasslands available for the experiment were one year in 2007, one and two years in 2008, one, two and three years in 2009 and one, two, three and four years in 2010. Each grassland was divided into two replicate blocks to which a split-plot design was applied with the main treatments (cut and unfertilized, cut and fertilized, grazed and unfertilized and grazed and fertilized) randomly allocated to four main plots. Subplot treatments consisted of three grassland mixtures (3-species, 10-species or 12-species mixtures). The 3-species mixture (3-mix) was composed of ryegrass, red clover and white clover, the 10-species mixtures (10-mix) had a further seven leguminous and non-leguminous forbs and the 12-species mixtures (12-mix) had in addition to the 10-mix two persistent species (Lucern and Festulolium) (Table 1). The size of the subplots was 5x9 m for the cutting treatment and 5x18 m for the grazing treatment. The seed rate of the mixture was 26 kg ha^-1^ and the seeds were sown at 0–1 cm depth. Climatic conditions were similar across the experimental years, with annual mean temperature ranging from 6.2 to 8.8°C and annual precipitation from 596 to 742 mm (Table A in S1 File). To avoid drought stress, the grasslands were irrigated in June and July of 2009 and in July 2010.

**Table 1:** Species included in the experiment, their functional group classification, the variety planted, and seed amount for the three mixtures.

Functional group	Species		Variety	1000 seed weight (g)	3-mix	10-mix	12-mix
					kg ha^-1^		
Grass							
	Perennial ryegrass	*Lolium perenne L*.	^1)^	2.7	21.3	17.2	7.4
	Festulolium	*Festulolium braunii K*.*A*.	Perun	3.7			8
Legume							
	White clover	*Trifolium repens L*.	^1)^	0.7	3.7	3	1.3
	Red clover	*Trifolium pratense L*.	Rajah	1.8	1	0.8	0.3
	Lucerne	*Medicargo sativa L*.	Pondus	2.1			4
	Birdsfoot trefoil	*Lotus corniculatus L*.	Lotanova	1.1		0.5	0.5
Forb							
	Chicory	*Cichorium intybus L*.	Spadona	1.5		0.7	0.7
	Plantain	*Plantago lanceolata L*.	^2)^	1.6		0.8	0.8
	Caraway	*Carum carvi L*.	Sylvia	2.9		0.8	0.8
	Salad burnet	*Sanguisorba minor Scop*.	^3)^	5.3		0.8	0.8
	Chervil	*Anthriscus cerefolium L*.	^3)^	2.2		0.6	0.6
	Sainfoin	*Onobrychis viciifoli Scop*.	^3)^	17.3		0.8	0.8

^1)^ Danish commercial mixture with perennial ryegrass and white clover (% of seed amount: 85% perennial ryegrass (30% medium tetraploid, 27% late diploid and 28% late tetraploid) and 15% white clover (11% large-leaved and 4% medium-leaved))

^2)^ Wild type

^3)^ Variety name not supplied.

In the fertilized plots cattle slurry was injected on the basis of total-N content. The dry matter (DM) content of the slurry was 3–7% and NH_4_-N was 56–66% of total-N. Due to the recycling of N under grazing, the fertilized grazed plots received 100 kg N ha^-1^ and the fertilized ungrazed cutting plots 200 kg N ha^-1^. The grazed plots were fertilized in spring, whereas the cut plots received 100 kg N in spring and 100 kg N following the first cut. The grazed plots were grazed continuously by pregnant heifers to approximately 5 cm stubble height. The mean number of grazing days per year was 1237 ha^-1^.

### Sample collection and analysis

In the cut plots, herbage was harvested with a Haldrup plot harvester (7 cm stubble height) from a 15 m^2^ subplot four times a year (early June, mid-July, late August and mid-October). In the grazed plots, herbage was harvested two times each year (2007–2009) corresponding to the first and third harvests under the cutting regime. The two harvest times were chosen because total biomass of the two periods accounted for almost 70% of annual biomass yield. Each time, newly fenced-off and recently topped subplots (20–35 m^2^) were selected for harvest. Biomass yield and botanical composition were determined for both the cut plots and the grazed plots. Botanical composition of the herbage in all harvested plots was determined in 200–500 g subsamples by hand-separation into individually sown and unsown plant species and dried at 80°C to constant dry weight. Perennial ryegrass and festulolium were pooled due to a severe difficulty in separating the two species in the lab. For chemical analyses of nutritive value, a 100-g sub-sample of herbage was collected from the three mixtures at each of the four cuts in 2007–2009 in the cutting treatment and oven-dried at 60°C. Ash was determined after combustion for six hours at 525°C, and nitrogen was determined according to the Dumas method [32]. Crude protein was calculated as total-N concentration (g kg^-1^) multiplied by 6.25. A Fiber-Tec system was used to determine neutral detergent fiber (NDF) [33]. In-vitro organic matter digestibility (IVOMD) was determined in rumen liquid [34].

### Statistical analyses

All data were analyzed using the R statistical language, version 3.2.3 [35]. Annual yield and botanical composition of the cutting treatment were analyzed in a linear mixed-effects model with species diversity, grassland age and slurry application as fixed factors and with calendar year (or field) and block as random factors. The botanical composition of forb and unsown species under the cutting treatment was log- or square-root transformed before analysis to obtain normality. Nutritive value was analyzed using a linear mixed-effects model with species diversity, grassland age, slurry application and cutting time as fixed factors, and calendar year and block as random factors. Tukey’s multiple comparisons were used to determine differences in nutritive value among the three mixtures. In order to compare the management effect (cutting vs. grazing) on yield production and botanical composition per cut, a linear mixed-effects model was applied using species diversity, grassland age, slurry application and management as fixed factors and calendar year, block and cut as random factors. Data of botanical composition of grass, legumes and forbs were square-root transformed before analysis in order to obtain normality. The Kruskal-Wallis test was conducted to analyze the effect of species diversity, grassland age, slurry application and management on botanical composition of unsown species, because the data of unsown species was not normally-distributed even after log or squared transformation.

## Results

### Dry matter productivity

**Fig 1:**
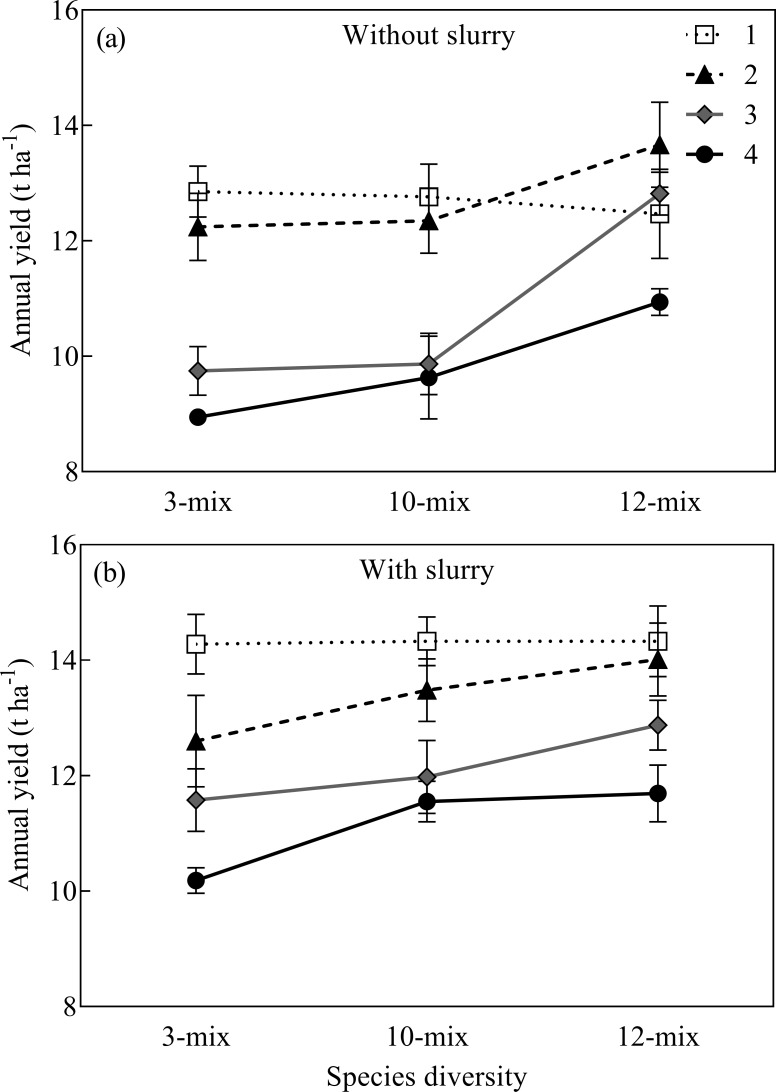
Annual yield (mean ± SE) of mixtures with increasing species diversity with and without slurry application of 1- to 4-yr-old grasslands.

Species diversity significantly increased the annual yield of the three mixtures under the cutting treatment (Fig 1, *P* < 0.0001), but this effect depended on grassland age and slurry application (Table B in S1 File, *P* < 0.05). Positive diversity effect on productivity was observed from the 2-yr-old grasslands onwards. The 12-mix generally had a significantly higher annual yield compared to the other two mixtures except that the significant difference in annual yield between 12-mix and 10-mix disappeared in the 4-yr-old grasslands (*P* < 0.05). Moreover, in the 3-yr-old grasslands the 10-mix significantly increased annual yield compared to the 3-mix with slurry application (*P* < 0.05). Overall, slurry application significantly increased the annual yield production of the three mixtures over four years by an average of 1.2 t ha^-1^ (*P* < 0.0001).

Annual yield production decreased with grassland age (Fig 1, *P* < 0.001), with this effect dependent on species diversity: the 12-mix showed sustained yields over four years without any statistically significant yield reduction (*P* = 0.66) while the annual yield of the 3-mix and the 10-mix significantly decreased with increasing grassland age (*P* < 0.0001 for both mixtures).

**Fig 2:**
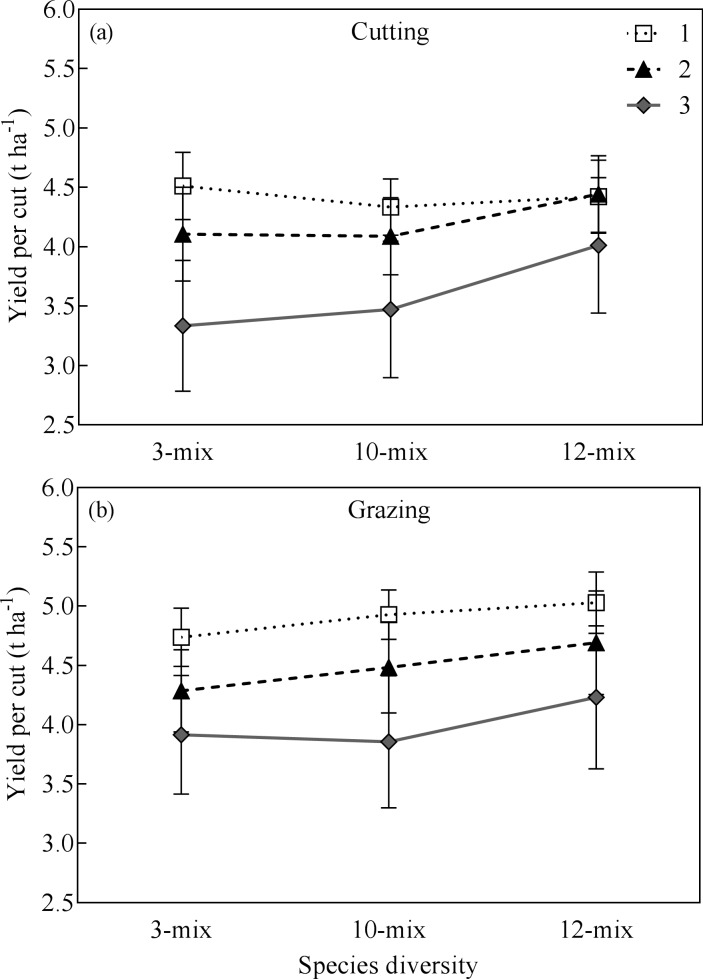
Yield (mean ± SE) per cut of mixtures with increasing species diversity under cutting and grazing management of 1- to 3-yr-old grasslands.

Annual herbage yields could not be calculated under the grazing regime, because only two cuts were made in temporarily fenced-off parts of the grazed plots at the same time as the cutting treatment in spring and August. Based upon herbage yield of the two cuts, we found a roughly 9% higher herbage yield under the grazing regime than under the cutting regime (Fig 2, *P* < 0.05). Regardless of regime, herbage yield per cut decreased with increasing grassland age (*P* < 0.001), but was greatly increased by slurry application (*P* < 0.0001). Notably, in contrast to the findings under the cutting regime, we found no difference in herbage yield per cut among the three mixtures under the grazing regime (*P* = 0.36).

### Botanical composition

**Fig 3:**
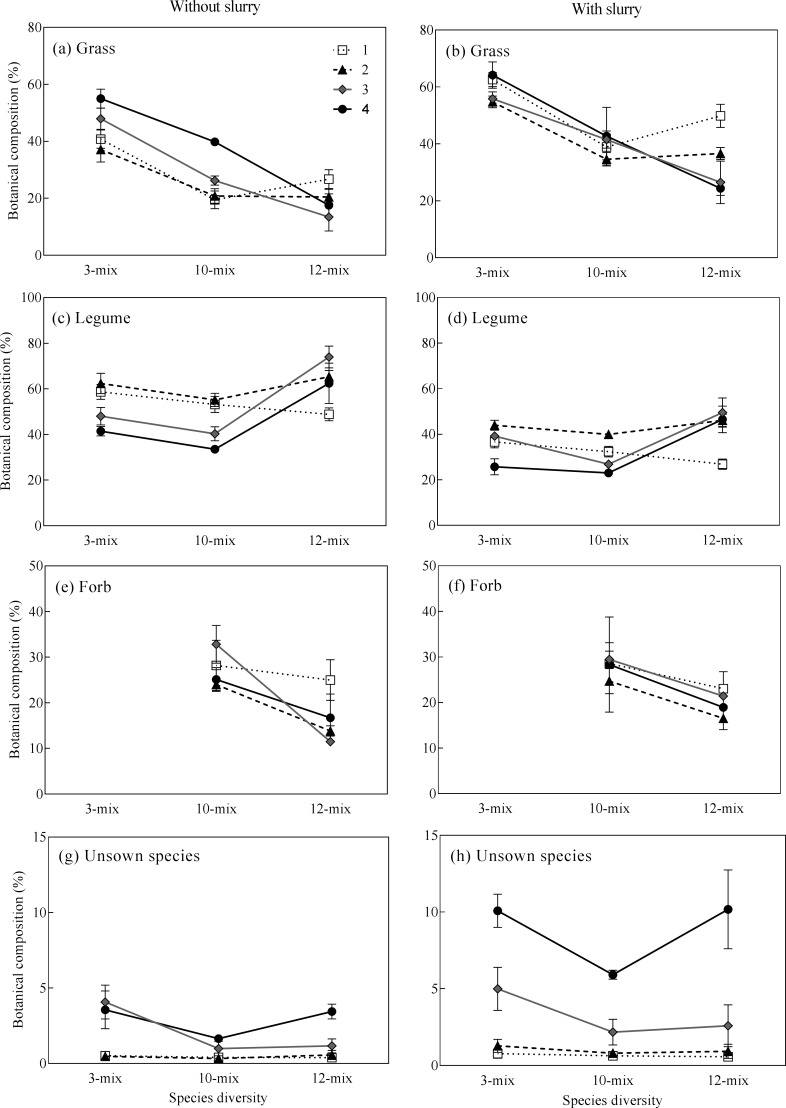
Botanical composition (% of DM) (mean ± SE) of different functional groups in three mixtures of 1- to 4-yr-old grasslands.

The three forage mixtures differed significantly in botanical composition of the three functional groups (Fig 3, grass: *P* < 0.0001, legume: *P* < 0.0001, forbs: *P* < 0.0001) and unsown species (*P* < 0.0001). The proportion of grass in the 3-mix was 19 and 25% higher than in the 10-mix and 12 mix, respectively (Fig 3A and 3B, *P* < 0.0001) and increased with grassland age (*P* < 0.0001). The 12-mix had a higher proportion of legumes and increased with grassland age (*P* < 0.0001), which was, respectively, 8 and 14% higher than the 3-mix and 10-mix (Fig 3C and 3D, *P* < 0.0001). The proportion of forbs was significantly higher in the 10-mix than the 12-mix by about 9% (Fig 3E and 3F, *P* < 0.0001). There were 1.6 and 0.7% more unsown species in the 3-mix than in the 10-mix and 12-mix, respectively (Fig 3G and 3H, *P* = 0.0001). Independent of the three mixtures, slurry application increased the proportion of grass (*P* < 0.0001) and unsown species (*P* = 0.001), decreased the proportion of legumes (*P* < 0.0001), but did not affect the proportion of forbs (*P* = 0.18).

**Fig 4:**
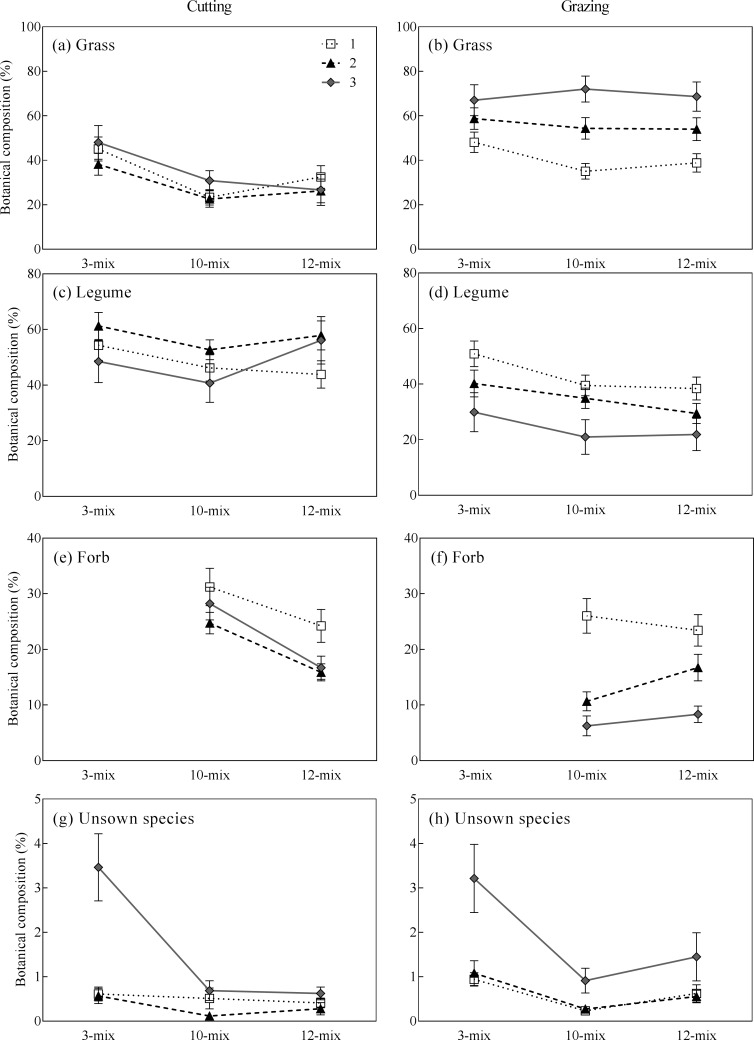
Botanical composition (% of DM) (mean ± SE) of different functional groups in three mixtures under cutting and grazing management of 1- to 3-yr-old grasslands.

There were significant differences between cutting and grazing in their effect on botanical composition of the three functional groups (Fig 4, grass: *P* < 0.0001, legume: *P* < 0.0001, forbs: *P* < 0.0001) and unsown species (Kruskal-Wallis chi-squared = 7.11, *P* = 0.008), with the difference depending on species diversity (grass: *P* < 0.05, and forbs: *P* < 0.0001) and grassland age (grass: *P* = 0.0001, legume: *P* = 0.0001, forbs: *P* < 0.0001). Overall, grazing resulted in a higher proportion of grass and unsown species but lower proportion of legume and forb species compared to the cutting treatment (Fig 4).

### Nutritive value

**Fig 5:**
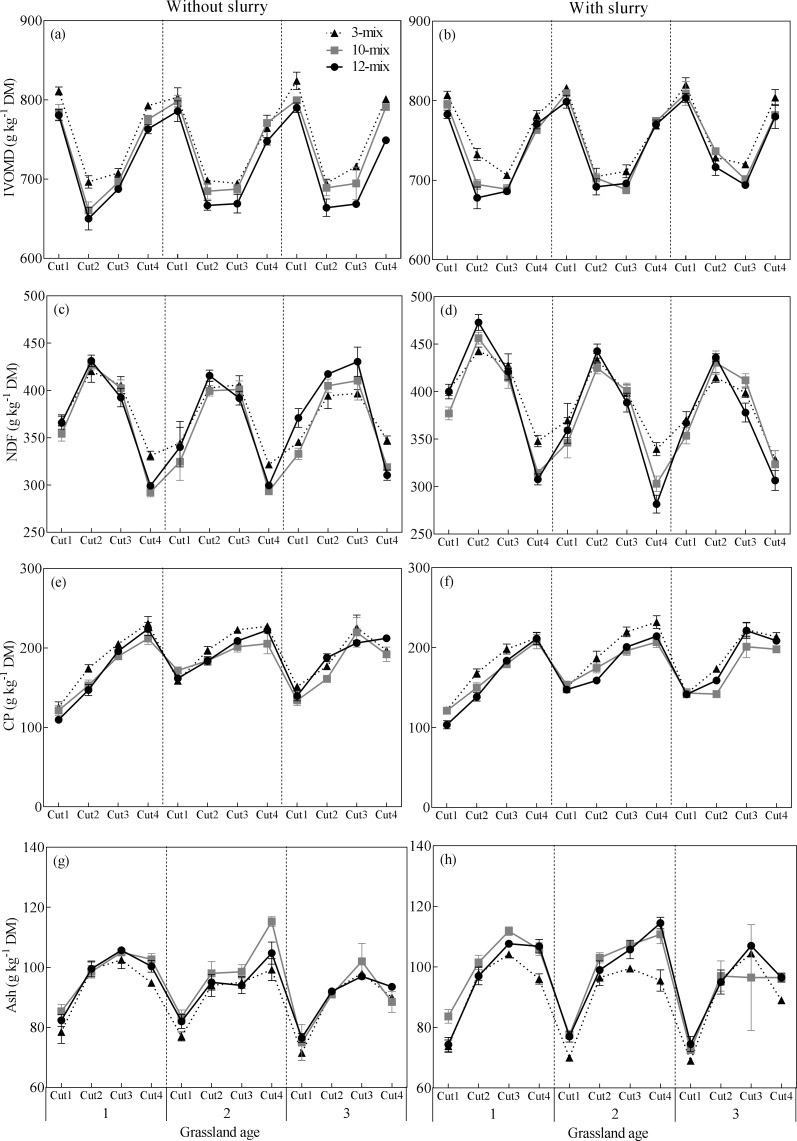
Interaction between forage mixture and time of cut on (a, b) in-vitro organic matter digestibility (IVOMD), (c, d) neutral detergent fiber (NDF), (e, f) Ash and (g, h) crude protein (CP) at different grassland ages. Data are mean ± SE.

The nutritive value of herbage was affected by species diversity, grassland age, cutting time and slurry application (Fig 5). There were significant interactions between species diversity and cutting time for NDF (*P* < 0.0001), ash (*P* < 0.01) and CP (*P* = 0.0001). Overall, the 3-mix had the highest concentrations of IVOMD, NDF, and CP (*P* < 0.001), while the 12-mix had the lowest IVOMD concentration (*P* < 0.001). The ash concentration was higher in the 10- and 12-mix than in the 3-mix (*P* < 0.001). The concentrations of NDF, ash and CP were not significantly different between the 10- and 12-mix.

The concentrations of IVOMD (*P* = 0.0001), NDF (*P* < 0.0001), CP (*P* < 0.0001) and ash (*P* < 0.0001) were significantly different in grassland age, but the direction and magnitude depended on cutting time (IVOMD: *P* < 0.05, NDF: *P* = 0.0021, Ash: *P* < 0.0001 and CP: *P* < 0.0001). IVOMD was higher in 3-yr-old grasslands than 2-yr-old grasslands (*P* < 0.01) but not different from the 1-yr-old grasslands. The concentrations of NDF and Ash were lower in 2- and 3-yr-old grasslands than in the 1-yr-old grasslands (*P* < 0.001). In contrast, CP was high in 2- and 3-yr-old grasslands than in the 1-yr-old grasslands (*P* < 0.001).

Slurry application significantly increased the concentrations of IVOMD (*P* < 0.0001) with this positive effect mainly observed on summer cut. Slurry application also significantly increased the concentrations of NDF (*P* < 0.0001) especially in the 1- and 2-yr-old grasslands and summer cut. Moreover, the concentrations of CP were higher when slurry was applied (*P* < 0.0001).

## Discussion

### The effect of diversity on productivity and persistence under cutting

Our study demonstrated that herbage productivity increased with richer species diversity under the cutting regime (Fig 1), which is consistent with previous studies on natural grasslands and forage mixtures [5, 36, 37]. The diversity-productivity relationship in natural grasslands has been explained by the complementarity of the species or by selection effects [5], but in this study the vigorous growth of lucerne in the 12-mix accounted for most of the yield increase from the second year onwards, especially in the unfertilized plots (Table C in S1 File). This suggests that the increase in yields with the higher species richness in the 12-mix was probably mainly due to a sampling effect. This is consistent with a previous study that showed that 6- and 9-species mixtures yielded more herbage than 2- and 3- species mixtures when including the highly productive legume, red clover [38]. In the present study there was no advantage conferred by the forb-rich 10-mix over the 3-mix in terms of annual biomass production when no slurry was applied–but there was when slurry was applied (Fig 1). This confirms that when including species with complementary traits to improve the species-richness of plant communities more resources can be accessed, enabling a rapid adaptation to climatic changes and more consistent productivity [39, 40].

The study demonstrated that species diversity enhances persistence of forage mixtures, evidenced by a consistently high yield in the most species-diverse mixtures (12-mix). In contrast, the 3-mix and the 10-mix showed significantly lower annual yields with increasing grassland age (Fig 1). The number of seeds gives a limit for establishment in the sward since, except for white clover, they cannot spread without seeding. The decreased perennial ryegrass content was consistent with the Jena experiment where reduced individual plant tiller numbers were the main cause [41]. Grassland of longer duration is an economically viable option in situations where the yield increase cannot pay for the costs of resowing the sward [42]. The persistence in yield production of the 12-mix in the current study reveals this as an option for future herbage production.

### The effect of diversity on nutritive value under cutting

The nutritive value of the forage mixture may depend on the dominant species, thus changes in botanical composition may affect the nutritive value of grasslands due to differences in the chemical composition and digestibility among the dominant species [43]. In the present study, the inclusion of several species of forbs or legumes in the sward affected the forage nutritive value. As the digestibility of organic matter (IVOMD) and NDF in grass were higher than in the forbs and legumes [16], IVOMD and NDF decreased when more forbs and legume species were included (Fig 5). Previous studies have shown that differences in nutritive value appear to be more closely related to differences in the relative proportions of the functional groups than to mixture complexity [16, 44]. We also found that the 10-mix and the 12-mix had similar nutritive value, while the 3-mix with a larger proportion of grass had the highest concentrations of IVOMD and NDF. It is surprising that the 3-mix also had a significantly higher CP than the other two mixtures. A previous study has shown that the CP concentration is controlled by the proportion of herbage legumes [16]. We found that the 12-mix had the highest proportion of legumes, but that this did not result in a higher CP concentration. The possible explanations are that (1) lucerne dominated in the 12-mix which has a lower CP concentration than white clover (Table E in S1 File) and (2) forbs had comparable CP concentrations to grass in the spring harvest but significantly lower CP concentrations than grass in the summer harvest. Thus, the 3-mix with a higher proportion of grass had an advantage in its CP concentration over the other two mixtures.

In a previous study, significant negative relationships were observed between nutritive value and the plant species richness of New Zealand pastures during summer and autumn growth [11]. Although increased species diversity resulted in greater stability of plant community biomass, it did not stabilize year-to-year variability of individual plant species abundances in natural grassland [45]. The plant species composition in the forage mixtures in the present experiment also changed dynamically with time under the different management regimes. We observed significant interaction of nutritive value and time of cut (Fig 5). Thus, time of cut should be taken into consideration when different forage mixtures are used, since the seasonal herbage quality varied among forage mixtures with different species compositions.

A concern regarding the use of complex forage mixtures is that variability in botanical composition may cause nutritive value to be unstable and ultimately affect animal performance [29]. However, even though forage mixtures may differ in some nutritive value components, the performance of feeding animals has been found not to be affected [46]. Furthermore, nutritive value typically decreases when plants mature. Thus, forage mixtures with more diverse species may have offset the plant maturity effects on nutritive value.

### The effect of diversity on productivity and botanical composition (cutting vs. grazing)

Grazing has profound effects on plant community composition and dynamic through several pathways, such as trampling, preference feeding and the recycling of N in animal excreta [47, 48], consequently affect the plant community productivity. Our study found that herbage production is higher under grazing compared with cutting. This effect could be caused by cattle preference feeding with legume and forb species resulting the dominance of grass species, which is consistent with a previous study reporting that the dominance of orchardgrass with a reduction of botanical composition of chicory and legume components in the forage mixture associated with a higher productivity [36].

Previous studies have shown a positive relationship between herbage yield and seeded species richness under the grazing regime. For example, pastures seeded with a mixture of 10 to 23 species of cool-season grasses and pasture herbs yielded more herbage that did simple perennial ryegrass-white clover mixtures [6, 7]. In the present study, however, the benefits of including a productive legume, lucerne, in the 12-mix in enhancing biomass production observed under the cutting regime disappeared under the grazing regime. That may be because continuous grazing restricts the ability for lucerne regrowth, which nearly eradicated it after three years (Table D in S1 File). Nevertheless, the 12-mix still tended to produce higher yield than the other mixtures (Fig 2), indicating that more diverse forage mixtures may contribute to more stable herbage yield under disturbance or stress because of species complementarity [36, 49, 50]. In addition, we found that forage mixtures with more diverse species had a lower proportion of unsown species than less diverse mixture under both cutting and grazing regimes (Fig 3G and 3H; Fig 4G and 4H). These findings are consistent with previous studies showing that plant species richness increases the resistance of ecosystems to weeds and other stresses [51–53].

## Conclusion

This study has shown the advantages of including other leguminous and non-leguminous forbs in short-term leys of perennial ryegrass, white clover and red clover. The species diversity increased sward productivity and persistence and the presence of one legume species, lucerne, considerably influenced yield under the cutting regime. Moreover, a greater species diversity in the forage mixture reduced invasibility by unsown species. However, these advantages depend on the cutting or grazing strategy since all forbs and legumes were reduced by continuous grazing. Our results reveal the importance of balancing dominant and non-dominant species to achieve higher yields without compromising the nutritive value. The main challenges are the choice of species and the number of seeds of the individual species in the seed mixture which is important to achieve high and stable yields and a high nutritive value in forage mixtures.

## Supporting Information

S1 File Supporting Methods and five tables. **Table A** Quarterly mean air temperature and precipitation over the experimental years. **Table B** Summary of statistics for linear mixed-effects models. **Table C** Botanical composition (% of DM) of three mixtures under cutting treatment with and without slurry application. **Table D** Botanical composition (% of DM) of three mixtures under cutting (C) and grazing (G) management. **Table E** Nutritive value of the single species and the functional groups averaged across slurry application in spring growth and summer (second) regrowth. IVOMD is shown in g kg^-1^ OM and the others parameters in g kg^-1^ DM.
(PDF)
