# Supplementary material for: Species Diversity Effects on Productivity, Persistence and Quality of Multispecies Swards in a Four-Year Experiment
Source: PLoS One. 2017 Jan 3;12(1):e0169208. doi: 10.1371/journal.pone.0169208 (PMC5207673; doi:10.1371/journal.pone.0169208)
Supplement: S1 File — Table A Quarterly mean air temperature and precipitation over the experimental years. Table B Summary of statistics for linear mixed-effects models. Table C Botanical composition (% of DM) of three mixtures under cutting treatment with and without slurry application. Table D Botanical composition (% of DM) of three mixtures under cutting (C) and grazing (G) management. Table E Nutritive value of the single species and the functional groups averaged across slurry application in spring growth and summer (second) regrowth. IVOMD is shown in g kg-1 OM and the others parameters in g kg-1 DM. (PDF) [file pone.0169208.s001.pdf]

S1 Appendix

Botanical composition of the herbage in all harvested plots was determined in 200-500 g subsamples by hand-separation into individual species and dried at 80°C to constant dry weight. Moreover, 80-100 g of each sown species was collected from the harvested material in spring and August cuts in the 12-mix cut-only plots in 2007-2009 and oven-dried at 60°C.

Table A. Quarterly mean air temperature and precipitation over the experimental years.

	2007		2008		2009		2010	
	^{o}C	mm	^{o}C	mm	^{o}C	mm	^{o}C	mm
Jan-Mar	3.8	189	3.6	200	1.5	108	-1.5	54
Apr-Jun	11.7	118	11.0	100	11.2	137	9.5	109
Jul-Sep	14.5	239	15.1	248	15.4	184	15.0	248
Oct-Dec	5.2	120	5.2	193	4.6	230	1.6	184

Table B. Summary of statistics for linear mixed-effects models.

**Model A**[1]		Mix	Age	Slurry	Mix *Age	Mix *Slurry	Age* Slurry	Mix*Age *Slurry
Annual yield (t ha^{-1})		***	***	***	***	NS	**	*
Botanical composition (%)	Grass	***	***	***	***	NS	**	NS
	Legume	***	***	***	***	NS	NS	NS
	Forb	***	NS	NS	**	NS	NS	NS
	Unsown species	***	***	**	*	NS	***	NS

**Model B**[2]		Mix	Age	Slurry	C/G	Mix*C/G	Age*C/G	Slurry*C/G
Yield per cut (t ha^{-1})		NS	***	**	*	NS	NS	NS
Botanical composition (%)	Grass	***	NS	***	***	*	***	NS
	Legume	**	*	***	***	NS	***	NS
	Forb	*	*	NS	***	***	***	NS
	Unsown species[3]	***	*	NS	**	NA	NA	NA

[1]Model A refers to the linear mixed-effects model, which was used to examine the main and interactive effects of species diversity (Mix), grassland age (Age) and slurry application (Slurry) on annual yield and botanical composition of grass, legume and forb under the cutting regime.
[2]Model B refers to the linear mixed-effects model, which was used to examine the main and interactive effects of species diversity (Mix), grassland age (Age), slurry application (Slurry) and cutting vs. grazing (C/G) on yield per cut and botanical composition of grass, legume and forb.
[3]Unsown species, the Kruskal-Wallis test was conducted to analyze the effect of species diversity, grassland age, slurry application and defoliation (cutting or grazing) on botanical composition of unsown species, because the data of unsown species violated the normal distribution assumption even after log or squared transformation. *** $P < 0.001$; ** $P < 0.01$; $P < 0.05$; NS, not significant; NA, not applicable.

Table C. Botanical composition (% of DM) of three mixtures under cutting treatment with and without slurry application.

Mix	Age	White clover		Red clover		Lucerne		Birdsfoot trefoil		Chicory		Plantain		Caraway		Burnet	
		0 N	200N	0 N	200N	0 N	200N	0 N	200N	0 N	200N	0 N	200N	0 N	200N	0 N	200N
3-mix	1	22.9	15.1	35.8	21.6												
	2	26.7	18.4	35.7	25.6												
	3	28.7	25.6	19.3	13.5												
	4	30.2	18.0	11.2	7.8												
10-mix	1	17.9	12.0	34.0	19.9			1.2	0.44	6.3	7.8	19.3	17.6	0.8	2.5	0.50	0.25
	2	23.8	14.5	31.1	25.4			0.29	0.08	9.9	11.1	9.1	6.3	4.7	7.1	0.04	0.07
	3	24.6	15.8	15.4	11.0			0.36	0.01	10.1	14.3	6.9	3.9	15.4	11.2	0.00	0.02
	4	25.6	15.4	7.9	7.7			0.10	0.00	6.0	8.4	10.6	2.3	8.4	17.7	0.02	0.00
12-mix	1	15.2	8.0	13.7	8.7	19.0	9.8	0.98	0.35	6.0	6.7	16.3	12.7	1.3	2.4	0.43	0.91
	2	14.6	10.1	11.2	7.6	39.4	28.3	0.18	0.16	5.4	8.1	5.5	3.8	2.6	4.3	0.07	0.13
	3	4.0	6.0	4.7	2.8	65.2	40.7	0.03	0.00	1.7	7.4	2.9	2.0	6.6	11.9	0.16	0.09
	4	7.2	5.4	2.1	2.1	53.0	39.0	0.16	0.00	1.9	7.8	4.9	1.4	9.6	9.7	0.19	0.00
Slurry		***		***		*		*		*		**		*		NS	
Mix		***		***		NA		NS		**		***		NS		NS	
Age		*		***		***		*		NS		***		***		*	
Slurry*Mix		NS		NS		NA		NS		NS		NS		NS		NS	
Slurry*Age		NS		NS		NS		NS		NS		**		NS		NS	
Mix*Age		***		NS		NA		NS		*		*		NS		NS	

Significance of main effects and interactions: ***P<0.001; **P<0.01; P<0.05; NS: not significant; NA: not applicable.

Table D. Botanical composition (% of DM) of three mixtures under cutting (C) and grazing (G) management.

Mix	Age	White clover		Red clover		Lucerne		Birdsfoot trefoil		Chicory		Plantain		Caraway		Salad burnet	
		C	G	C	G	C	G	C	G	C	G	C	G	C	G	C	G
3-mix	1	17.1	18.3	37.2	32.7												
	2	21.2	29.7	40.0	10.4												
	3	25.1	26.8	23.4	3.0												
10-mix	1	11.6	14.3	33.4	24.4			1.3	0.9	7.6	10.3	20.7	13.2	1.2	1.0	0.5	0.6
	2	15.9	21.7	36.6	12.9			0.2	0.2	9.4	5.1	8.5	2.7	6.6	2.6	0.1	0.1
	3	19.7	18.4	20.5	2.4			0.6	0.0	6.2	1.4	5.7	0.6	15.7	4.2	0.1	0.0
12-mix	1	8.9	12.3	15.7	18.4	18.3	6.4	0.9	1.4	6.3	9.1	15.4	12.1	0.9	0.7	0.7	0.2
	2	9.8	20.3	10.5	7.5	37.3	0.8	0.2	0.9	5.8	8.3	5.7	3.0	4.0	4.1	0.2	0.4
	3	6.5	19.3	6.9	2.1	42.7	0.2	0.1	0.1	3.0	3.5	3.7	0.7	9.7	4.1	0.2	0.1
C/G		**		***		***		NS		NS		***		***		NS	
Mix		***		***		NA		NS		NS		**		NS		NS	
Slurry		**		***		NS		**		NS		**		**		NS	
Age		**		***		NS		**		***		***		***		NS	
C/G*Mix		NS		*		NA		**		*		*		*		NS	
C/G*Slurry		NS		NS		NS		NS		NS		NS		*		NS	
C/G*Age		NS		***		***		NS		**		NS		***		NS	

Significance of main effects and interactions: ***P<0.001; **P<0.01; P<0.05; NS: not significant; NA: not applicable.

Table E. Nutritive value of the single species and the functional groups averaged across slurry application in spring growth and summer (second) regrowth. IVOMD is shown in g kg^{-1} OM and the others parameters in g kg^{-1} DM.

	Species								Functional groups			Significance Species			Significance Functional groups		
	WC[1]	RC	LU	BT	CI	PL	CA	SB	GR[2]	Leg	Forbs	Sp.	N	Sp.*N	Group	N	Gr.*N
Spring																	
IVOMD	815^{b}	790^{c}	712^{e}	730^{d}	842^{a}	728^{d}	833^{a}	610^{f}	831^{Aa}	764^{B}	767^{B}	*	NS	NS	*	NS	NS
NDF	219^{f}	265^{d}	340^{b}	267^{d}	235^{e}	338^{b}	277^{d}	301^{c}	425^{Aa}	273^{B}	285^{B}	***	NS	NS	***	NS	NS
ADF	193^{f}	198^{ef}	270^{a}	219^{d}	203^{e}	275^{a}	236^{c}	256^{b}	242^{Ac}	220^{B}	242^{A}	***	**	NS	*	NS	NS
ADL	26^{d}	24^{de}	48^{b}	44^{bc}	22^{e}	55^{a}	26^{d}	40^{c}	18^{Bf}	34^{A}	35^{A}	***	NS	NS	***	NS	NS
CP	213^{a}	193^{b}	204^{b}	221^{a}	105^{c}	103^{cd}	100^{de}	97^{e}	88^{Bf}	208^{A}	101^{B}	***	NS	*	***	NS	NS
Ash	105^{a}	84^{c}	88^{c}	89^{c}	114^{b}	89^{c}	105^{a}	88^{c}	62^{Cd}	92^{B}	100^{A}	***	***	NS	***	NS	NS
Summer																	
IVOMD	732^{b}	704^{c}	629^{d}	630^{d}	684^{c}	575^{e}	817^{a}		743^{Ab}	677^{B}	692^{AB}	***	NS	NS	*	NS	NS
NDF	304^{e}	342^{d}	402^{c}	364^{d}	352^{d}	466^{b}	236^{f}		517^{Aa}	352^{B}	351^{B}	***	NS	NS	***	NS	NS
ADF	273^{d}	265^{d}	335^{b}	321^{b}	298^{c}	370^{a}	230^{e}		320^{b}	300	300	***	NS	NS	NS	NS	NS
ADL	49^{b}	39^{c}	66^{a}	71^{a}	38^{c}	65^{a}	30^{d}		25^{Ce}	55^{A}	44^{B}	***	NS	NS	***	NS	NS
CP	235^{a}	193^{c}	213^{b}	212^{b}	134^{f}	118^{g}	161^{e}		177^{Bd}	213^{A}	138^{C}	***	NS	NS	***	NS	NS
Ash	102^{d}	87^{e}	81^{e}	81^{e}	133^{b}	109^{c}	162^{a}		114^{Bc}	88^{C}	135^{A}	***	NS	NS	***	NS	NS

1) WC: white clover, RC: red clover, LU: lucerne, BT: birdsfoot trefoil, CI: chicory, PL: plantain, CA: caraway, SB: salad burnet, GR: grass; ADF: acid detegent fiber, ADL: acid detegent lignin.

2) Grass includes perennial ryegrass and festulolium. Grass is both included in the statistical calculations of species and functional groups

Different capital letters within functional groups indicate significant differences (P<0.05)

Different lower case letters within species indicate significant differences (P<0.05)

Significance of main effects and interactions: ***P<0.001; **P<0.01; P<0.05; NS: not significant
